# A low-cost, portable device for the study of the malaria parasite’s growth inhibition via microwave exposure

**DOI:** 10.1016/j.ohx.2024.e00540

**Published:** 2024-06-06

**Authors:** Esteban Rua, Lorena Coronado, Carlos A. Donado Morcillo, Ricardo Correa, Lina Solís, Carmenza Spadafora, Alejandro Von Chong

**Affiliations:** a School of Electrical Engineering, Universidad Tecnológica de Panamá, Víctor Levi Sasso Campus, Panama City, Panama; b Sistema Nacional de Investigación-SENACYT, Panama City, Panama; c Biomedical Physics and Engineering Unit, Center of Cellular and Molecular Biology of Diseases (CBCMe), Instituto de Investigaciones Científicas y Servicios de Alta Tecnología (INDICASAT AIP), Panama City, Panama

**Keywords:** Irradiation system, Malaria, Plasmodium, Microwaves

## Abstract

Recently, a novel method for the growth inhibition of malaria parasites using microwaves was proposed. However, the apparatuses used to demonstrate this method are high-cost and immovable, hindering the progression in this field of research, which is still in its early stages. This paper presents the redesign, construction, and validation of an equivalent system, converting it into a portable and low-cost system, capable of replacing the existing one. The proposed system is mainly composed of an RF generator (MAX2870), an RF amplifier (SKYWORKS 66292-11) and a graphical user interface. Likewise, the RF applicator proposed by the original study was redesigned, resulting in a five-fold improvement in return loss. The obtained results indicate that the proposed system achieves 90% parasite growth inhibition, matching the performance of its counterpart at less than 1% of its cost. These results represent a breakthrough for the creation of smaller, enhanced devices that open new possibilities for an alternative treatment to combat this devastating disease.

## Graphical abstract

**Figure:**
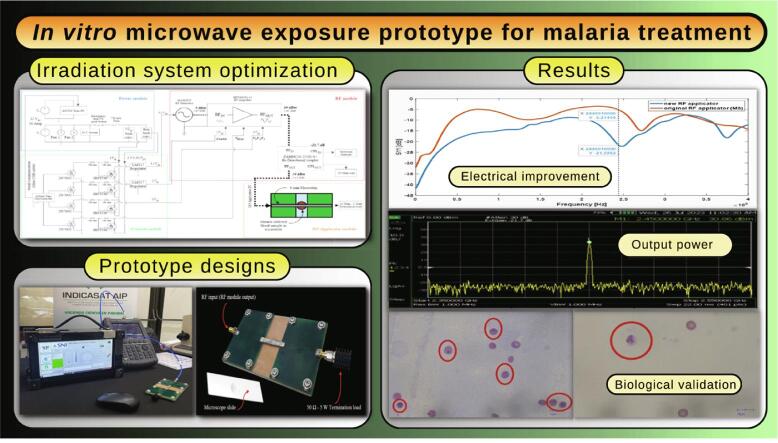

**Specifications table**.

**Table:** 

Hardware name	In Vitro System for Malaria Growth Inhibition via Microwave Exposure
Subject area	• Engineering and materials science • Biological sciences (e.g., microbiology and biochemistry) • Educational tools and open-source alternatives to existing infrastructure
Hardware type	• Measuring physical properties and in-lab sensors • Biological sample handling and preparation • Field measurements and sensors • Electrical engineering and computer science
Closest commercial analog	No commercial analog is available.
Open source license	MIT License
Cost of hardware	∼USD 950
Source file repository	https://zenodo.org/records/10211082
OSHWA certification UID	PA000002

## 1 Hardware in context

Malaria is a life-threatening disease caused by parasites that are transmitted by female anopheles mosquitoes. Only in 2021, 240 million infections were reported, causing more than six hundred thousand deaths [1]. According to [2], antimalarial drug resistance has become a growing concern in countries susceptible to malaria outbreaks. Due to the increasing resistance of the malaria parasites against traditional drugs and the lack of an optimal vaccine, the search for a new approach has become critical [3]. To the best of our knowledge, the first attempt deviating from a pharmaceutical approach began in [4], when it was proposed to inhibit the growth of the malaria parasite through controlled exposure to fixed electric fields. Unexpectedly, exposing malaria parasites to DC electric fields contributed to their proliferation rather than their inhibition, yet it also meant that the parasites reacted to external electrical stimulation. Nonetheless, in [5] it was shown that when using microwaves rather than a DC stimulus, the proliferation of the malaria parasites decreased significantly.

**Fig. 1:**
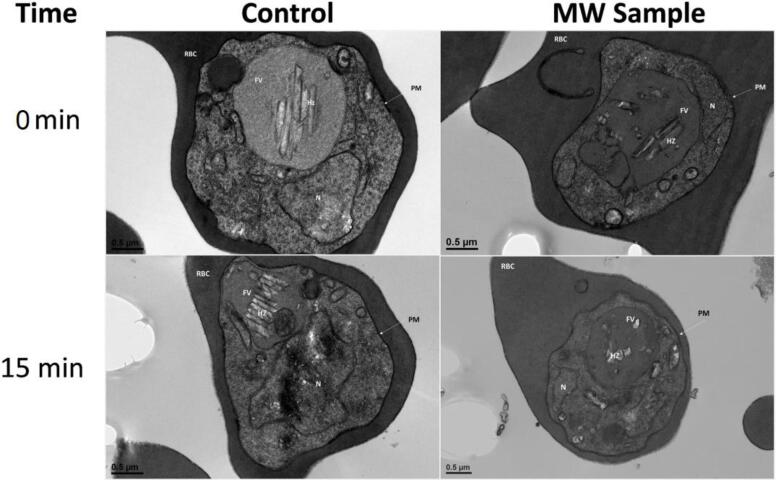
Ultrastructural changes in infected erythrocytes (i.e., red blood cells) immediately after (0 min) and 15 min after microwave treatment. On the left is the control (unexposed cells) and on the right the cells exposed to microwaves (MW Sample) [5].

To date, the exact mechanism on how microwaves inhibit malaria proliferation is still unknown. A possible hypothesis states that when the malaria parasite infects an erythrocyte (i.e., a red blood cell), the hemoglobin present in it is degraded into a byproduct (heme), which is toxic to the parasite. The parasite neutralizes this byproduct by turning it into a crystal known as hemozoin, which is paramagnetic. The interaction of an infected erythrocyte with electromagnetic fields (at a specific frequency), results in the destruction of the hemozoin crystals present in them, releasing the heme. The heme release causes oxidative stress to the parasite, leading to apoptosis (i.e., programmed cell death). In their study, Coronado et al. also determined that the novel approach was selective for malaria parasites, leaving mammalian cell lines unaffected. Fig. 1 shows the comparison between the structural changes in erythrocytes in a non– irradiated blood sample (control) versus an irradiated blood sample. One might initially presume that the inhibition is caused by a thermal effect, however the authors of this study also ruled out this hypothesis.

The physical setup that the research team used to demonstrate the effectiveness of this method comprises standard equipment ubiquitous in RF laboratories, mainly an RF generator (HP E4433A, Hewlett-Packard, CA, United States) and an RF amplifier (OPHIR 5171FE, OPHIR RF, CA, United States) controlled via a Graphic User Interface (GUI) [5]. Despite the effectiveness and reliability that characterize this setup, it is a high-cost, heavy and voluminous system, hindering the progression of this research. Considering that the study of the interactions between electromagnetic radiation and cellular behavior requires highly specialized equipment, normally unavailable in a single laboratory, designing a portable device to perform these tests is of enormous value.

Apart from the RF equipment, an additional device to irradiate the blood samples is needed. For this purpose, the research group developed two RF applicator models. Initially, a resonant cavity prototype applicator was designed, in which a microtube with a 100 μL sample was inserted, then irradiated at 2.45 GHz. This prototype reached an average inhibition effectiveness of 90 %, but the power required for its operation is high (12 W) [6]. Seeking a more efficient applicator in terms of required power, an applicator known as M3 (Maternal Malaria & Malnutrition) was created subsequently. The M3 consists of a microstrip transmission line engraved on an FR4 PCB board, which has a substrate cutout in the middle and a microstrip line (bridge) connecting the two sides. In this substrate cutout, a 50 uL sample of contaminated blood is placed under the microstrip bridge using a microscope slide. The M3 achieved the same parasite inhibition effectiveness as the resonant cavity prototype, yet at 1 W. However, the original design could be improved, since it has a return loss of 3.045 dB at 2.45 GHz, resulting in significant reflected power.

## 2 Hardware description

This work proposes a compact, lightweight, and low-cost generator device intended to replace the existing generator system. Our primary contribution lies in the detailed delineation of materials and methodologies used to construct this enhanced, low-cost generator system, along with a redesigned RF applicator with improved return loss. From a hardware perspective, these advancements not only facilitate the development of future prototypes but also critically advocate for refining the existing prototype to advance research in this emergent treatment modality with life-saving potential. From a biological perspective, the availability of an equivalent yet portable generator system enables further investigation into the underlying mechanisms of this phenomenon, which is crucial for transitioning this method into real-world clinical applications.

The hardware is composed of four interconnected modules.

1. The RF generator module
2. The power module
3. The control module
4. The RF applicator module

### 2.1 Component selection

For the proposed device, the target operation parameters were taken from the original study, which are an RF signal at a frequency of 2.45 GHz and power of 1 W (30 dBm). These parameters yielded the best results in terms of parasite growth inhibition [5]. Table 1 shows a list of commercially available components that meet the design requirements for this task.

**Table 1:** Commercially available components for the proposed device.

**Component**	**Manufacturer**	**Model**	**Output freq (2.45 GHz)**	**Power output (1 W)**	**Evaluation board**	**Cost**
**RF Generator**	Analog Devices	MAX2870	**✓**	N/A	**✓**	LOW
	SeeSii	LibreVNA 2.0	**✓**	N/A	**✓**	HIGH
	Mini-Circuits	ISC-2425–25+	**✓**	N/A	**✓**	VERY HIGH
**RF Amplifier**	Skyworks Solutions	SKY66292-11	**✓**	**✓**	**✓**	LOW
	Mini-Circuits	ZVE-8GX+	**✓**	**✓**	**✓**	VERY HIGH
	Skyworks Solutions	SE2576L	**✓**	**✓**	✘	VERY LOW

The selection criteria for these components were primarily based on cost and ease of implementation. From Table 1, all the RF generators listed can generate the desired output frequency of 2.45 GHz. Among these, the MAX2870 from Analog Devices (Massachusetts, United States) was chosen for its lower cost and simpler implementation. It features commercially available evaluation boards that can be powered ON/OFF directly using a microcontroller, unlike the other options, which are more expensive and require external software for control. Regarding the RF amplifier, the SKY66292-11 from Skyworks Solutions (Massachusetts, United States) is not only cost-effective but also simpler to implement compared to the SE2576L from the same manufacturer. This is due to the availability of an evaluation board for the SKY66292-11. It is important to note that all selected components have an impedance of 50 Ω to ensure maximum power transfer and to increase return loss.

### 2.2 RF generator module

**Fig. 2:**
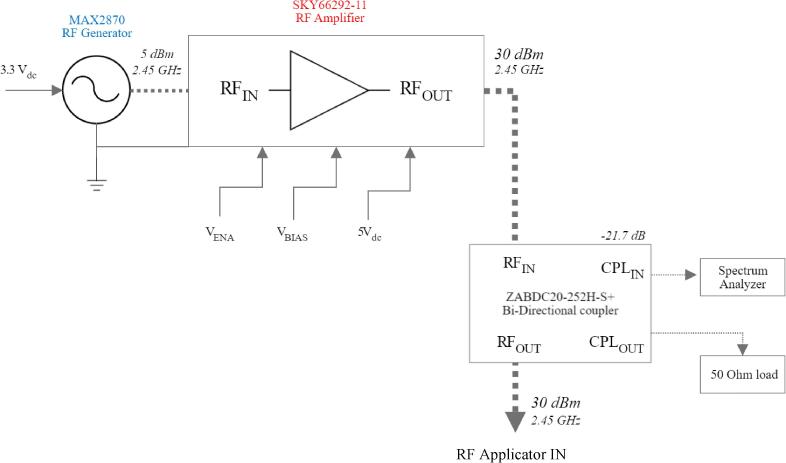
RF generator module block diagram.

Central to the proposed device is the RF generator module, which comprises the RF generator, the RF amplifier, and the directional coupler. A block diagram of the RF generator module can be seen in Fig. 2.

For the RF signal generation, an evaluation board based on the MAX2870 integrated circuit was used. It operates using two voltage-controlled oscillators (VCOs) which, when combined with an external reference oscillator and a loop filter, make it a high- performance frequency synthesizer capable of achieving frequencies from 23.5 MHz to 6 GHz. This evaluation board can be powered by a 5 V micro-USB connector or by a 3.3 V direct power supply.

The RF amplifier is an evaluation board based on the SKY66292-11 integrated circuit. The SKY66292-11 is a power amplifier (PA) operating in a frequency range of 2.3 GHz to 2.4 GHz, with an output power of up to 36 dBm (4 W) [7]. This amplifier offers a high output power for its size and cost, which optimizes the construction of the prototype. The evaluation board has a 2 V enable input, a bias input and three amplification stages which must be powered with 5 VDC. The bias input is supplied with a voltage ranging from 4.75 to 5.25 V to compensate for the voltage drops across the three amplifier stages. When using this amplifier, a turn-on and turn-off sequence must be followed to avoid damaging the device. Although this amplifier was designed to operate at frequencies between 2.3 GHz and 2.4 GHz, it can operate satisfactorily at frequency of 2.45 GHz. It is worth highlighting that 2.45 GHz is an open frequency, belonging to the ISM band (Industrial, Scientific & Medical) [8]. Thus, this device can be used without the need for a specific license or permission.

The RF generator module uses a Mini-Circuits ZABDC20-25H + four-port directional coupler, designed to provide an attenuated sample from the amplifier for safe measurement by a spectrum analyzer. The coupler is utilized to measure the power sent to the RF applicator. A Pasternack PE6150 termination load, rated at 1 W and 50 Ω, was selected for its maximum VSWR of 1.1:1 across a DC to 4 GHz bandwidth. The connection of the RF generator module components can be seen in Fig. 2 (in this image, the dotted line indicates a microwave signal). Connections between components are made by using SMA male to SMA male cables.

### 2.3 Power module

The primary role of this module is to energize the other constituent modules of the device. It incorporates a 12 V-10 A power supply from ALITOVE, which features two separate 12 V channels. One channel is dedicated to powering the mini-PC, display, and fans, while the other serves the remaining electronic components of the device. The mini-PC, sourced from AWOW (Shenzhen, China), consumes a maximum current of 3 A and is responsible for hosting the GUI that facilitates system control via serial communication with the control module. The forced ventilation system, essential for maintaining consistent airflow across the device, consists of two 12 V fans: the Artic F9 Silent (Switzerland) and the WINSINN model (China). Since one of the objectives of this design was to make it portable, two different fans were used to comply with the available space.

**Fig. 3:**
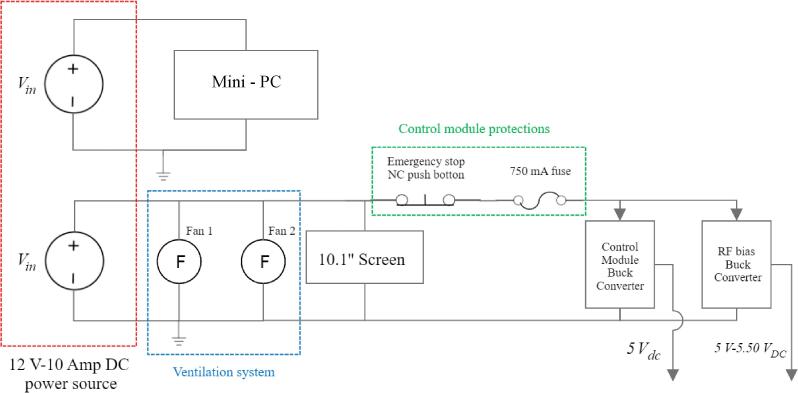
Power module block diagram.

A 750-mA fuse is integrated to safeguard equipment connected to the second channel of the power supply, including the RF and control modules. A normally closed emergency stop button is added to allow for abrupt system shutdown if required. The emergency stop button disconnects the RF generator module only. Abrupt shutdown of the mini-PC via the emergency button is not required since it would damage the mini-PC. The system's GUI is relayed via a display, specifically a 10-inch touchscreen manufactured by SunFounder (Shenzhen, China). A schematic representation of the power module is illustrated in Fig. 3.

### 2.4 Control module

**Fig. 4:**
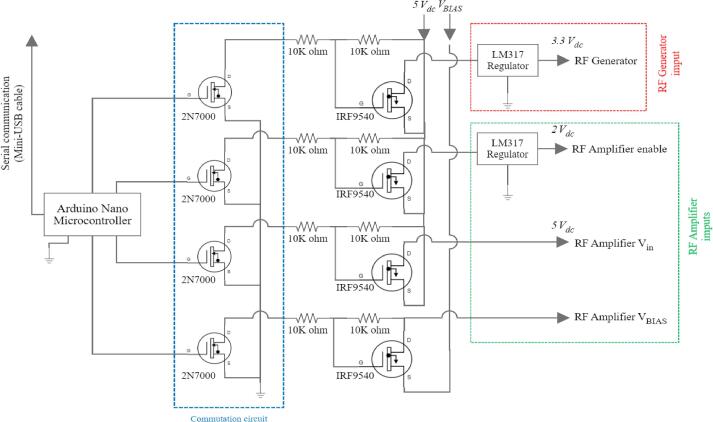
Control module schematic.

The control module governs the activation and deactivation of the RF generator module devices, responding to user-defined parameters set via the GUI. This module encompasses two buck converters, an Arduino Nano microcontroller, and a custom-designed switching and regulation circuit. A schematic of the control module is depicted in Fig. 4.

Buck converters are included to transform the primary supply voltage to a level that is compatible with the various electronic equipment. The primary converter steps down the 12 V source voltage to 5 V, which powers the RF amplifier, the RF generator, and the Arduino Nano microcontroller. The secondary converter is used to modulate the bias input voltage of the amplifier, ensuring voltage drop compensation for optimal device performance. The bias voltage, adjustable between 4.75 V and 5.25 V, should be finely tuned to consistently maintain 5 V in the amplifier's power stages during normal operation.

The Arduino Nano microcontroller interfaces with the mini-PC and the GUI through serial communication, enabling the activation and deactivation of the RF generator module based on user action. This control mechanism turns on and off the RF generator module using MOSFETs 2 N7000 and IRF9540, via the Arduino Nano's digital pins. The system incorporates four distinct switching subcircuits, each linked to a microcontroller's digital pin.

1. The first subcircuit is used for the RF amplifier’s enable pin, set at 2 V, by incorporating an LM317 voltage regulator connected to the power transistor’s drain.

2. The second subcircuit is responsible for the amplifier's power stage pins (3), operating directly at 5 V without necessitating regulation.

3. The third subcircuit is for enabling the bias voltage of the power amplifier.

4. The fourth subcircuit is used for turning the RF Generator on/off via the 3.3 V input. For the voltage step down, a LM317 is used.

### 2.5 RF applicator module

The RF applicator is the final component of the proposed irradiation device. It is based on a microstrip transmission line, composed of three sections: the strip, the substrate material, and the ground plane (GP). The operation of this transmission line is like that of a parallel plate capacitor, with the strip and ground plane being the electrodes (both 1 oz copper compounds), with the substrate material acting as a dielectric.

As a design objective, the proposed RF applicator needed to be redesigned to increase its electrical efficiency (i.e., increasing return loss or decreasing S11), while maintaining the same inhibition effectiveness as the original. This is important to reduce costs, by using smaller electrical components and by increasing the number of simultaneous experiments that can be run concurrently in a lab setup (growing cultures is a slow process). Running experiments in parallel is critical to obtain statistically significant results.

**Fig. 5:**
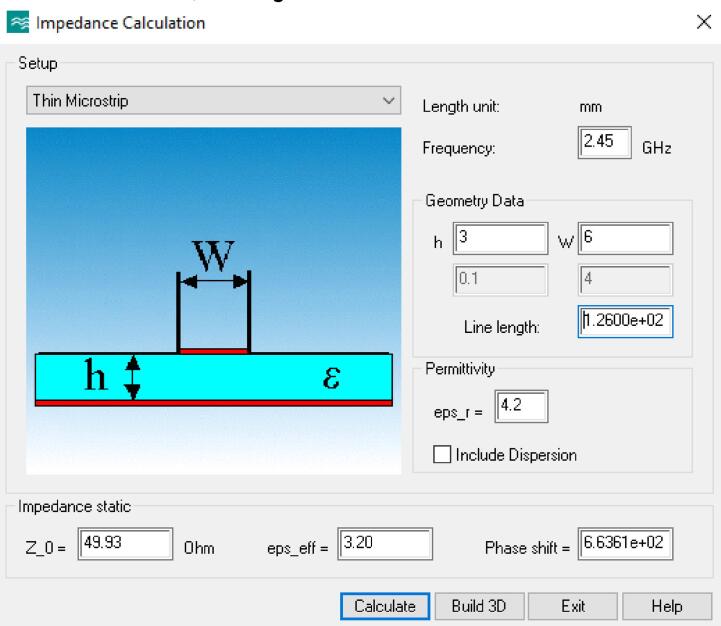
Transmission Line Impedance Analytical Calculator in CST Studio Suite Student Edition.

The impedance of a transmission line is mainly defined by the height of the substrate, the width of the strip and the relative permittivity of the substrate material. In this case, FR4 PCB boards were chosen, which is composed mainly of fiberglass. This material has a relative permittivity of 4.2 (dimensionless) and an effective permittivity of 3.2. The combination of these parameters yields a net impedance of approximately 49.93 Ω. Since all components have a 50-ohm impedance, the microstrip line was designed to be of this impedance to ensure impedance matching. The modeling of the RF applicator was carried out using CST Studio Suite Student Edition software, see Fig. 5 and Table 2.

**Table 2:** Design parameters for the proposed RF applicator.

**Parameter**	**Notation**	**Value**
*Frequency*	f	2.45 GHz
*Wavelength*	λ	122.45 mm
*Substrate height*	h	3 mm
Microstrip width	W	6 mm
Line length	l	126 mm
Relative permittivity	$\varepsilon_{r}$	4.2
Effective permittivity	$\varepsilon_{\mathrm{eff}}$	3.2
Phase shift	φ	66.36°
Line impedance	$Z_{o}$	49.93 Ω (VSWR = 1.001)

The calculated line impedance was 49.93 Ω (VSWR = 1.001) which is very close to the ideal value of 50 Ω (VSWR = 1). However, this line impedance is an approximation. The software calculates the net impedance of the transmission line assuming a uniform microstrip (without discontinuities). To insert the microscope slide (with the blood sample to be irradiated) under the microstrip, a substrate cutout of 75 mm x 25 mm x 3 mm was made in the middle of the proposed RF applicator.

The substrate cutout height was chosen as 3 mm since the blood drop must not touch the microstrip, as it would degrade the microstrip material’s integrity. The air gap between the blood sample and the microstrip should be minimized to enhance interactions with the fields near the strip conductor, but it should also be considered that the physical properties of blood can vary from patient to patient (varying the blood’s meniscus height once placed on the microscope slide).

In practice, the variation of the S11 and the impedance of the device is minimal. The air gap is filled with a Schott Foturan II slide (a material composed mainly of glass and ceramics). The combined effect of the slide and the blood droplet is similar to that of the FR4 material, and this is demonstrated by calculating the equivalent dielectric constant of the central space in the RF applicator. With an FR4 gap of 0.5 mm (ε*_r_* = 4.4), a length of 1 mm corresponding to the slide (ε*_r_* = 5.5), a drop of blood with a height of 1 mm (ε*_r_* = 75) [5], [9], [10] and an air gap of 0.5 mm (ε*_r_* = 1.0006) the equivalent dielectric constant is obtained by the following calculation:

$$\varepsilon_{\mathrm{eq}}=\frac{h_{\mathrm{cavityheight}}}{\frac{h_{\mathrm{air}}}{\varepsilon_{\mathrm{air}}}+\frac{h_{\mathrm{blood}}}{\varepsilon_{\mathrm{blood}}}+\frac{h_{\mathrm{slide}}}{\varepsilon_{\mathrm{slide}}}+\frac{h_{FR4}}{\varepsilon_{FR4}}}$$

$$\varepsilon_{\mathrm{eq}}=\frac{3mm}{\frac{0.5mm}{1.0006}+\frac{1mm}{75}+\frac{1mm}{5.5}+\frac{0.5mm}{4.4}}$$

$$\varepsilon_{\mathrm{eq}}\approx 3.71$$

With this dielectric constant, an updated line impedance of 52.78 Ω (VSWR = 1.056) was obtained, which represents a behavior similar to a microstrip transmission line without substrate cutout (S11 measurements are presented in the validation section).

To assess transmission line losses, the RF applicator's loss tangent — the ratio of the imaginary to the real part of relative permittivity — was analyzed. Ideal loss tangent values are near 0, indicating negligible losses, with values below 0.01 being acceptable [11]. To compute the RF applicator's equivalent loss tangent at 2.45 GHz, loss tangent values for materials in its hollow cavity were used: tan(δ) = 0.27 for blood [5], [9], [12], tan(δ) = 0.02 for FR4 [13], and tan(δ) = 0.02 for the microscope slide [14]. Air, having a loss tangent of nearly 0, is excluded from this calculation. The equivalent loss tangent is calculated using the individual loss tangent values of the materials in the RF applicator's hollow cavity.

$${\tan\delta }_{\mathrm{eq}}=\frac{1}{\frac{1}{{\tan\delta }_{\mathrm{blood}}}+\frac{1}{{\tan\delta }_{\mathrm{slide}}}+\frac{1}{{\tan\delta }_{FR4}}}$$

$${\tan\delta }_{\mathrm{eq}}=\frac{1}{\frac{1}{0.27}+\frac{1}{0.02}+\frac{1}{0.02}}$$

$${\tan\delta }_{\mathrm{eq}}\approx 3.71$$

**Fig. 6:**
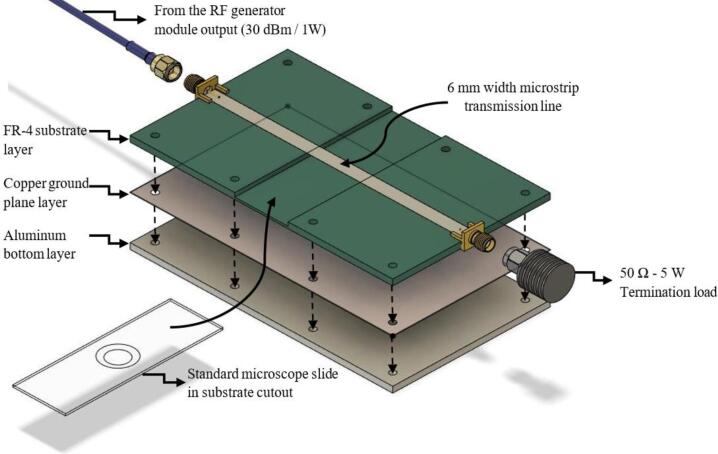
3D model of the RF applicator.

As the loss tangent is below 0.01, it indicates that the RF applicator maintains good overall system efficiency. The RF applicator model can be seen in Fig. 6.

The RF applicator has two Mueller SMA PCB mount (Germany) welded at each end of the microstrip. At one end the output of the directional coupler is connected while the other has a Pasternack PE6095 termination load (50-ohm). A tin coating is applied to the microstrip to protect it from oxidation processes. The manufacturing process of this module is explained in section 5.

When constructing the RF applicator, it is advisable to place an aluminum layer for increased mechanical support, a more robust ground plane and protecting it from electromagnetic interference. For the proposed applicator, an aluminum sheet of 3 mm thick was used.

### 2.6 Modules assembly

**Fig. 7:**
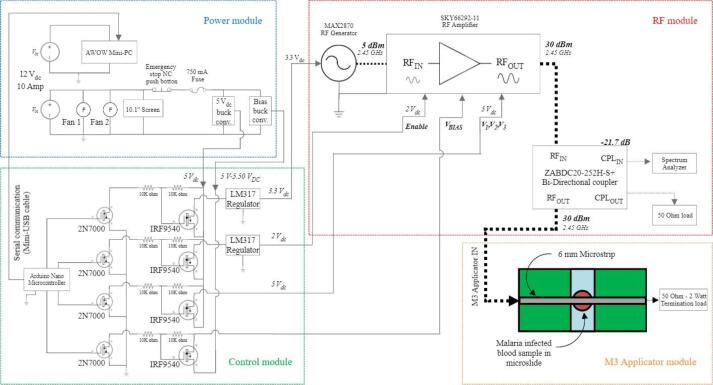
Schematic of the four modules assembled.

After connecting the four modules described in the previous sections, the resultant assembly contains both the irradiation system prototype and the in vitro applicator (see Fig. 7).

## 3 Design files

Wiring diagrams, PCB layouts, and Gerber files for PCB manufacturing were generated using the EasyEDA design software.

The coding files for the Arduino Nano microcontroller (INO files) were developed using the Arduino IDE. The graphical interface was developed with NI LabVIEW. The.vi extension files are also included in this section.

The Gerber files for cutting and engraving RF applicator parts can be found at Microstrip_engraving_model.zip.

The steps to follow for the construction of the RF applicator are in section 5.2.

The 3D models were produced with Autodesk Fusion 360 software. All STL files can be found within 3D_printable_support_structures.zip and the editable version in 3D_modificabledesign.f3d. Laser cutting models for exterior acrylic parts can be found in Acrylics_laser_cutting.zip. For assembly instructions, refer to section 5.4. Design files are shown in Table 3.

• Control_circuit_wiring_diagram: electrical connections of the prototype’s control system circuit. It contains the connections of the control module and the interconnections with the other modules (JSON file).
• Control_circuit_PCB_layout: layout of the prototype’s control system circuit PCB (JSON file).
• Control_circuit_PCB_manufacturing: files required for manufacturing the primary-side module’s PCB (GTL, GBL, DRL).
• RF_Applicator_parts_engraving: files required for manufacturing the RF applicator. Section 5.2 explains the order in which the pieces should be cut.
• Acrylics_laser_cutting: contains the drawings for the laser cutting in SVG format.
• 3D_printable_support_structures: contains all the STL files for 3D printing.
• Microstrip_engraving_model: editable file that contains the trajectories for the RF applicator manufacturing.
• ControlCircuitArduinoCode: code to upload to the Arduino Nano microcontroller.
• ExposureSystemSoftware: LabVIEW source code for the graphical user interface.
• SystemAssembly: irradiation prototype final build assembly instructions.

**Table 3:** Design files summary.

**Design file name**	**File type**	**Open source license**
*Control_circuit_wiring_diagram*	ZIP file	*MIT license*
*Control_circuit_PCB_layout*	ZIP file	*MIT license*
*Control_circuit_PCB_manufacturing*	ZIP file	*MIT license*
*RF_Applicator_parts_engraving*	ZIP file	*MIT license*
*Acrylics_laser_cutting*	ZIP file	*MIT license*
*3D_printable_support_structures*	ZIP file	*MIT license*
*Microstrip_engraving_model*	ART file	*MIT license*
*ControlCircuitProgram*	INO file	*MIT license*
ExposureSystemSoftware	ZIP file	*MIT license*
SystemAssembly	PDF file	*MIT license*

## 4 Bill of materials

In addition to the list of electronic components used for the construction of the irradiation device (Table 4), another table containing the equipment needed for the manufacturing process was included in Table 5. These devices are commonly found in electrical engineering or manufacturing laboratories.

**Table 4:** Bill of materials.

**Designator**	**Component**	**Number**	**Cost per unit − USD**	**Total cost −** **USD**	**Source of materials**	**Material type**
MAX2870-EVB	MAX2870 based RF signal generator evaluation board (23.5 to 6000 MHz)	1	46.46	46.46	Amazon	Non-specific
SKY66292-11-EVB	SKY66292-11 based RF power amplifier evaluation board (4 W, 2.2 to 2.4 GHz)	1	105.19	105.19	DigiKey Electronics	Non-specific
ZABDC20-252H+	21.7 dB Bi-Directional Coupler, 800–––2500 MHz, 50 Ω		151.73	151.73	Mini-Circuits	Non-specific
Mini-PC	Mini Desktop Computer Intel Celeron J3455 Windows 10 Pro, 8 GB DDR4/128 GB SSD	1	125.99	125.99	Amazon	Non-specific
Touch screen	10 Inch Touch Screen for Raspberry Pi 10.1″ HDMI 1280x800 IPS	1	139.99	139.99	Amazon	Non-specific
Arduino Nano	Arduino Nano development board	1	12.99	12.99	Amazon	Non-specific
Buck converters	LM2596 DC to DC Buck Converter 3.0–40 V to 1.5–35 V Power Supply Step Down Module	1	8.99	8.99	Amazon	Non-specific
Forced draft fan	F9-92 mm Standard Case Fan (12 V, 1800 RPM)	1	7.99	7.99	Amazon	Non-specific
Induced draft fan	WINSINN DC 60 mm Fan 12 V 6010 Double Ball Bearing Brushless Cooling 60mmx10mm 2PIN	1	10.99	10.99	Amazon	Non-specific
Emergency button	Emergency Stop Push Button Switch (AC 660 V, 10A)	1	11.99	11.99	Amazon	Non-specific
Fuse	5TT 750-R – 750 mA, 250 V AC DC fuse cartridge	1	0.62	0.62	DigiKey Electronics	Aluminum
Fuse holder	BF303 fuse holder –15 A, 500 V	1	2.07	2.07	DigiKey Electronics	Non-specific
R_1_, R_2_, R_3_, R_4_, R_5_, R_6_, R_7_, R_8_	Carbon Film Resistors – Through Hole 10 K Ohm ¼ W 5 % 250 V	8	0.10	0.80	DigiKey Electronics	Carbon film
R_9_	Carbon Film Resistor – Through Hole 220 Ohm ¼ W 5 % 250 V	1	0.10	0.10	DigiKey Electronics	Carbon film
R_10_	Carbon Film Resistor – Through Hole 330 Ohm ¼ W 5 % 250 V	1	0.10	0.10	DigiKey Electronics	Carbon film
R_11_	Carbon Film Resistor – Through Hole 680 Ohm ¼ W 5 % 250 V	1	0.10	0.10	DigiKey Electronics	Carbon film
R_12_	Carbon Film Resistor – Through Hole 120 Ohm ¼ W 5 % 250 V	1	0.10	0.10	DigiKey Electronics	Carbon film
C_1_	Aluminum Electrolytic Capacitor – Radial Leaded 0.1uF 50 V	1	0.27	0.27	Digikey Electronics	Aluminum
C_2_	Aluminum Electrolytic Capacitor – Radial Leaded 1uF 50 V	1	0.24	0.24	Digikey Electronics	Aluminum
C_3_, C_4_	Aluminum Electrolytic Capacitors – Radial Leaded 10uF 35 V	2	0.28	0.56	DigiKey Electronics	Aluminum
Q_1_, Q_2_, Q_3_, Q_4_	2 N7000 MOSFETs –N-CH 60 V 200 mA TO92-3	4	0.52	2.04	DigiKey Electronics	Semiconductor
FET_1_, FET_2_, FET_3_, FET_4_	IRF9540 MOSFETs –P-CH 100 V 19 A TO220AB	4	2.15	8.60	DigiKey Electronics	Semiconductor
MOSFET heatsinks	Heat Sink TO-220 Aluminum 3.0 W @ 80 °C Board Level	2	0.75	1.50	DigiKey Electronics	Aluminum
U_1_, U_2_	LM317T-DG adjustable regulator –IC 1.5 A TO220-3	2	0.95	1.90	DigiKey Electronics	Semiconductor
Fixed terminal blocks	Fixed Terminal Blocks 2 Poles, Screw Type, 5.0 Pitch	8	0.66	5.28	DigiKey Electronics	Non-specific
Plastic acrylic sheets	Clear acrylic plexiglas sheet, pack of 2 (8x12x1/4in)	1	12.99	12.99	Amazon	Acrylic
Breadboard jumper wires	120 x Multicolored MM, MF, FF Breadboard Jumper Wires	1	7.99	7.99	Amazon	Non-specific
Dupont connector headers	Male Header Pins, 40-Pin Header Strip (2.65 mm)	1	4.99	4.99	Amazon	Non-specific
DC power connector	Male 12v DC Power Jack Adapter Connector	1	5.99	5.99	Amazon	Non-specific
Mini-USB cable	USB 2.0 A to Mini B 5-pin cable	1	7.99	7.99	Amazon	Non-specific
HDMI cable	One foot HDMI cable	1	5.98	5.98	Amazon	Non-specific
SMA cables	5-Pack 6 Inch SMA Male to Male Coax Coaxial Cable	1	11.99	11.99	Amazon	Non-specific
Power chord pigtail	18 Gauge 3 Prong 120 V, 10 A Power Supply Cord	1	9.99	9.99	Amazon	Non-specific
Carrying handle grip	Generic 20Cm PVC Carrying Handle	1	7.56	7.56	Amazon	Non-specific
Coupler termination load	1 Watt RF Load Up to 4 GHz with SMA Male Gold-Plated Brass	1	8.89	8.89	Pasternack	Aluminum
RF applicator termination load	5-Watt RF Load Up to 18 GHz With SMA Male Input Black Anodized Aluminum Heatsink	1	159.99	159.99	Pasternack	Aluminum
SMA PCB mounts	SMA Connector Jack, Female Socket 50Ohm Board Edge, End Launch Solder	2	4.99	9.98	Mouser Electronics	Metal
Microscope slides	Eisco Labs Microscope Slides, Single Conv, Pack of 10	1	8.99	8.99	Amazon	Glass
Copper clad PCB (single side)	Single Side Copper Clad Laminate PCB Circuit Board FR4 150x100mm 5.91x3.94 in. 1.5 mm Thickness	1	8.99	8.99	Amazon	Copper
PCB solder mask	UV Curable Solder Mask PCB BGA Repair Paint	1	8.19	8.19	Amazon	Non-specific
Aluminum plates	General Purpose Plate 1/8″ (3 mm) x 2″ (50 mm) Long 0.118″ Aluminum Square Flat Bars 6061	1	21.99	21.99	Amazon	Aluminum
**TOTAL**				**949.08**		

**Note:** the cost of the 3D-printed pieces was not included in this bill of materials.

**Table 5:** Bill of supplementary devices.

**Designator**	**Component**	**Number**	**Cost per unit − USD**	**Total cost −** **USD**	**Source of materials**	**Material type**
3D printer	R QIDI TECHNOLOGY 3D Printer High Precision Printing (10.6x7.9x7.9 in)	1	699.00	699.00	Amazon	Non-specific
Laser-cutting machine	Glowforge Basic 3D laser printer	1	2995.00	2995.00	Glowforge	Non-specific
Milling machine	Bantam Tools desktop CNC milling machine	1	3900.00	3900.00	Bantam tools	Non-specific
Soldering station	SMD and through hole regulable soldering station	1	139.99	139.99	Amazon	Non-specific
Vector-network analyzer	LibreVNA 2.0 100 kHz-6 GHz Vector Network Analyzer	1	699.08	699.08	Amazon	Non-specific
Spectrum analyzer	2023 Upgraded TinySA Ultra Spectrum Analyzer, 4.0 Inch SeeSii, 100 kHz to 5.3 GHz	1	259.99	259.99	Amazon	Non-specific
UV lamp	10 W High power UV led lamp for spaces IP65	1	16.99	16.99	Amazon	Non-specific

## 5 Build instructions

### 5.1 Control module manufacturing

**Fig. 8:**
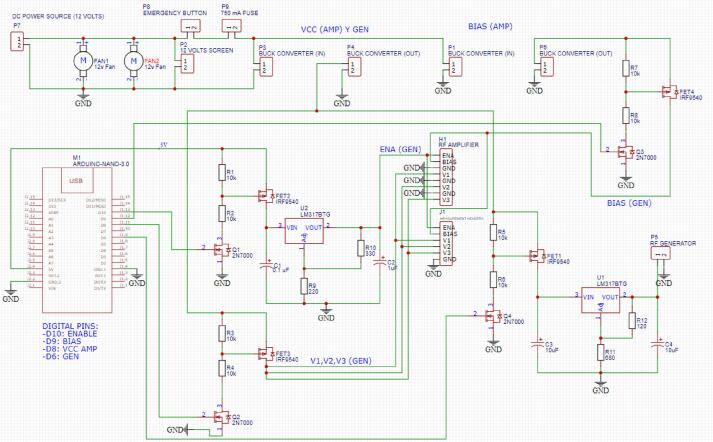
Control module schematic.

Fig. 8 illustrates the wiring diagram for the device’s control circuit (found in Control_circuit_wiring_diagram.pdf). We strongly recommend to manufacture a printed circuit board (PCB) using a CAM-CAD design software (such as EasyEDA), and a milling machine (a 100 x 70 mm FR4 PCB size is adequate). The circuit’s diagram can be opened directly with any design software uploading the file **Control_circuit_wiring_diagram.json**. The PCB layout is available in **Control_circuit_PCB_layout.json**. The Gerber files needed to print the control circuit can be generated from the diagram; likewise, those present in the **Control_circuit_PCB_manufacturing** folder can be used directly. For this design, a 0.8 mm bit can be used for track milling and hole drilling.

**Fig. 9:**
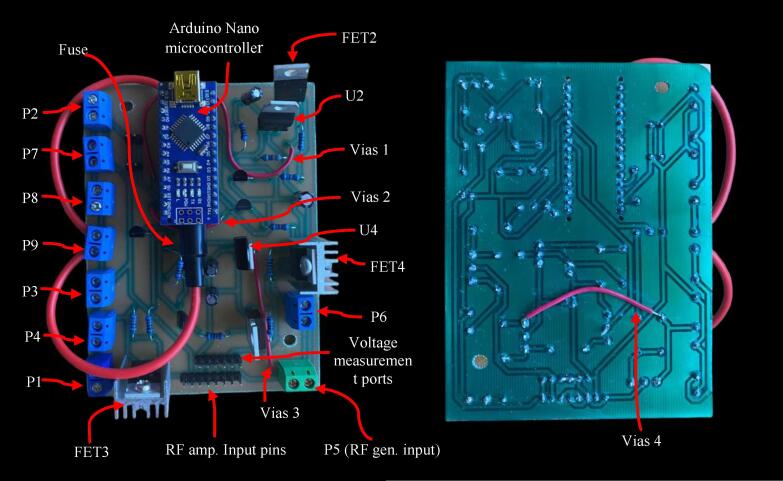
Control module PCB. Top (left) and bottom (right) views.

The assembly of the control circuit components can be seen in Fig. 9. We also recommend applying a layer of UV solder mask before soldering the components of the control circuit to protect it from wearing and preventing short circuits. Finally, the PCB was manufactured using single-sided copper clad to simplify the design. The heat sinks for power MOSFET transistors are placed in FET3 and FET1. As these transistors switch the generator and amplifier on and off, they are more prone to heating up.

### 5.2 RF applicator module manufacturing

**Fig. 10:**
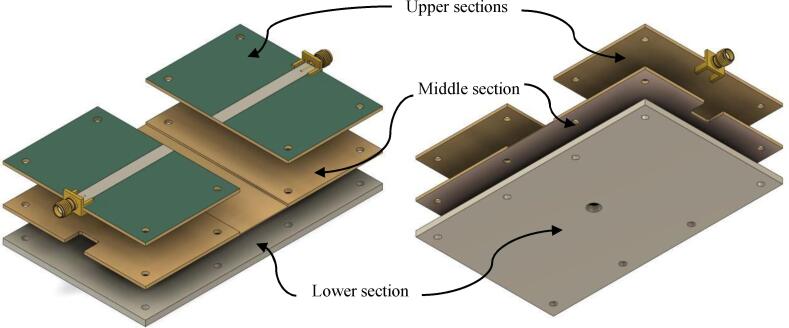
Top (left) and bottom (right) view of RF applicator sections.

Like the control module circuitry, the RF applicator was also manufactured using a Bantam Tools CNC milling machine and single-sided copper clad plates. Each of these copper clad plates has a thickness of 1.5 mm, so, to achieve the required thickness of 3 mm, it was necessary to place two of these plates on top of each other. The distribution of copper clad plates is shown in Fig. 10.

The upper section of the RF applicator consists of two microstrip sections derived from engraving and cutting a copper clad plate measuring 150 mm x 100 mm x 1.5 mm. The manufacturing steps for the upper section are:

1. Set the dimensions of the copper clad plate in the CNC milling machine software and select a 2 mm drill bit. The bit sizes are based on the Gerber codes for the RF applicator but can be altered in the Microstrip_engraving_model.art file to generate a new custom Gerber code.
2. Secure the copper clad onto the CNC bed with the copper face upwards and the FR4 face against the cutting bed.
3. Execute the Microstrip_copper_cleaning.tap file from the RF_Applicator_parts_engraving folder in the CNC software. This removes excess copper, leaving a central 6 mm wide strip. It's crucial to use the bit corresponding to the Gerber code, as a different size would produce a strip with incorrect dimensions, affecting the RF applicator's performance due to impedance mismatch.
4. After removing the copper, a 6 mm wide microstrip should remain. Without moving it from the CNC bed, run the PCB_screw_holes.tap file to create screw holes for assembling the RF applicator.
5. Run the Microstrip_superior_sections.tap file to divide the plate into two sections, each 50.2 mm x 75.6 mm x 1.5 mm, creating a space of 25.3 mm x 75.6 mm x 2 mm for the slide.
6. Apply solder mask to the microstrip sections, leaving a 5 mm strip exposed for soldering the SMA PCB mounts. Use acetate paper for a uniform finish.
7. Solder the central pin of an SMA PCB mount to one end of each microstrip section. Attach the ground pins of the SMA port to the ground plane in the lower section.

The lower section, made of a single-sided FR4 copper sheet, is attached to the upper sections. Its manufacturing steps are:

1. Set the dimensions of the copper clad plate in the CNC software and select a 2 mm drill bit, with the option to modify the Gerber code parameters in the Microstrip_engraving_model.art file.
2. Properly align the copper clad on the CNC bed with the copper face downwards and the FR4 face upwards.
3. Run the PCB_screw_holes.tap program to create screw holes for assembling the RF applicator.
4. Execute the SMA_mount_hole.tap program to prepare the lower section for the SMA PCB mount, ensuring symmetry.
5. Insert a 1 mm bit and run the PCB_microscope_hole.tap program to carve a 1 mm diameter hole at the edge of the microstrip for observing the erythrocytes.
6. Replace with a 2 mm bit and execute the Microscope_slide_central_hole.tap program to create a 2 mm deep cavity for the slide and blood drop.

An aluminum sheet, 3 mm thick, adds mechanical rigidity and magnetic insulation to the RF applicator. Its manufacturing steps are:

1. Insert a 3 mm thick aluminum sheet, sized to the RF applicator, into the engraving machine.
2. Use a 3 mm drill bit and run the Aluminum_microscope_hole.tap program to drill screw holes and a 6 mm diameter hole aligned with the 1 mm hole in the lower section.

The final assembly involves:

1. Stacking the upper and lower sections.
2. Soldering the lower SMA PCB mount pins to the copper of the lower section.
3. Placing these sections on the aluminum sheet.
4. Securing the layers with eight 3 mm diameter, 10 mm height screws, using washers for even pressure distribution. Proper fastening is crucial to avoid performance-reducing air gaps.
5. Fastening the screws on the applicator's bottom face with 3 mm nuts.
6. Cutting a 6 mm wide, 30 cm long copper tape section and tinning it to prevent oxidation.
7. Aligning the tinned tape with the upper section's microstrips and welding it. Ensuring straightness is vital to avoid power reflections.

**Fig. 11:**
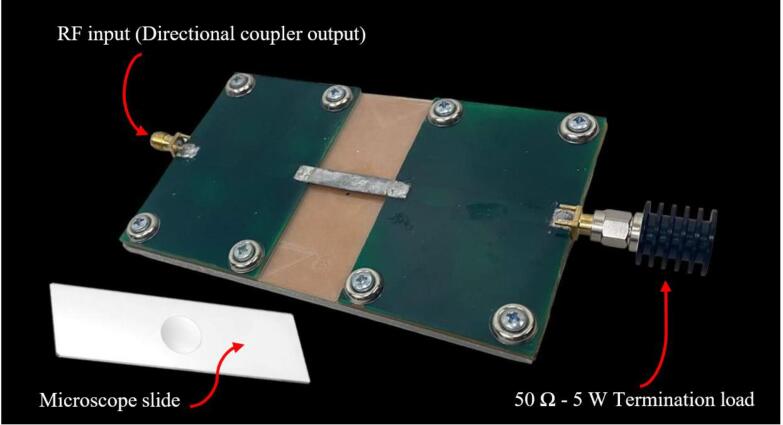
Manufactured RF applicator.

The completed RF applicator is illustrated in Fig. 11.

One end is connected to the output of the directional coupler, while the other end is connected to a termination load of 50 Ω. This will preserve maximum power transfer throughout the irradiation system.

### 5.3 3D printed supports and case manufacturing

Once the RF applicator is built, the parts for the assembly of the device are cut and printed. As for the 3D pieces, they should be printed in the quantities shown in Table 6:

**Table 6:** System’s 3D printed supporting pieces.

**Support piece file**	**Description**	**Number**	**Material**
Border_joint1.stl	Parts to join the sections of the acrylic box.	7	TPU
Border_joint2.stl	Parts to join the sections of the acrylic box.	1	TPU
Control_circuit_support	Part to fix the control circuit and RF generator inside the acrylic box.	1	PLA / ABS
_piece.stl			
Coupler_support.stl	Part to fix the directional coupler to the acrylic box.	1	PLA / ABS
Handle_support_piece1	Part to fix the luggage bag in the acrylic box.	1	PLA / ABS
.stl			
Handle_support_piece2	Part to fix the luggage bag in the acrylic box.	1	PLA / ABS
.stl			
MiniPC_support.stl	Part to fix the Mini-PC to the acrylic case.	1	PLA / ABS
Screen_support.stl	Parts to fix the screen to the acrylic box.	3	PLA / ABS

To 3D print the support structures, load the STL files located in 3D_printable_support_structures.zip in a printing software (the QIDI printing software can be downloaded at http://www.qd3dprinter.com/software/). Next, configure the preferences for every piece and save the file to a removable drive. Plug the drive to the printer, set up the printer and start printing. If you have another 3D printer you can upload the files to any other laminator software.

The manufacturing of the acrylic box was carried out by cutting 6 mm thick acrylic sheets in a laser cutter from the manufacturer Glowforge. For that, the files present in the Acrylics_laser_cutting.zip folder are used. For the laser cutting, load the files located in Acrylics_laser_cutting.zip in a laser cutting software (Glowforge software is available at https://app.glowforge.com/). Carefully introduce the acrylics in the printer slot, close the lid and wait for the software to align the designs. Once the piece is aligned, start cutting. When the printing is finished, open the lid, and slowly remove the acrylics. For safety reasons, do not open the lid while the printer’s laser is still operating. Additionally, it is recommended to keep the laser cutter near a window or connected to an extractor, since the fumes produced can be harmful to health.

### 5.4 System assembly

**Fig. 12:**
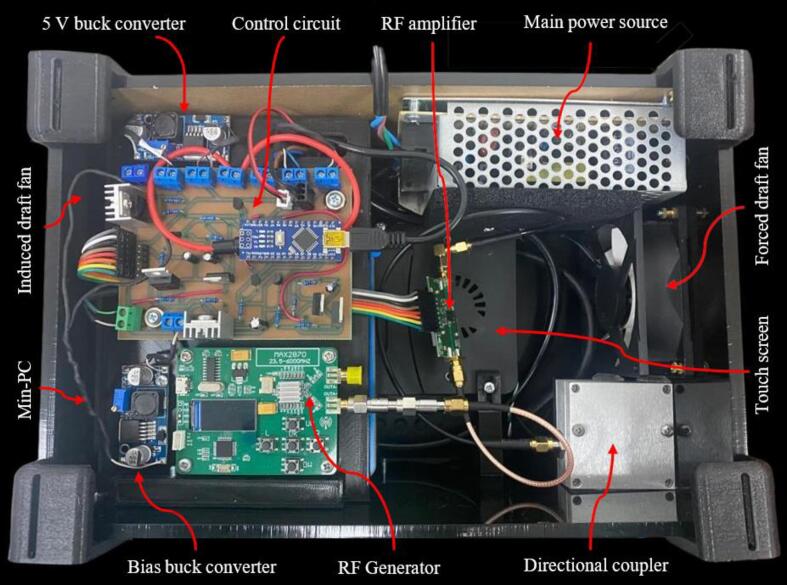
Irradiation prototype internal distribution.

**Fig. 13:**
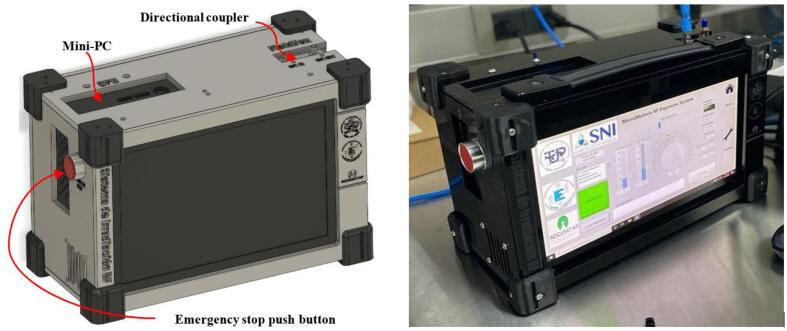
Irradiation system final assembly (left: 3D model, right: device built).

Once all the PLA and acrylic parts have been printed, the system can be assembled. In the SystemAssembly.pdf contains the instructions to assemble the device. Fig. 12 shows the internal arrangement of the irradiation prototype. Fig. 13 shows the assembled device.

## 6 Operation instructions

### 6.1 Arduino IDE code setup

To load the control circuit program, follow these steps:

1. Download and install Arduino IDE from: https://www.arduino.cc/en/software.
2. Copy and paste the ‘‘Libraries” folder from the repository to the Arduino IDE folder created after the installation.
3. Open Arduino IDE.
4. Open ‘‘ControlCircuitArduinoCode.ino” in Arduino IDE.
5. Connect Arduino Nano to the computer using a miniUSB cable.
6. In Arduino IDE, navigate to Tools > Board: Select ‘‘Arduino Nano”.
7. Click ‘‘upload” and wait for the code to upload and wait for the ‘‘upload completed” message.

### 6.2 LabVIEW program setup

1. Access to the following website and select your PC specifications: https://www.ni.com/es- cr/support/downloads/software-products/download.LabVIEW.html
2. Download and install LabVIEW.
3. Run ExposureSystemSoftware.vi.

### 6.3 LabVIEW GUI description

**Fig. 14:**
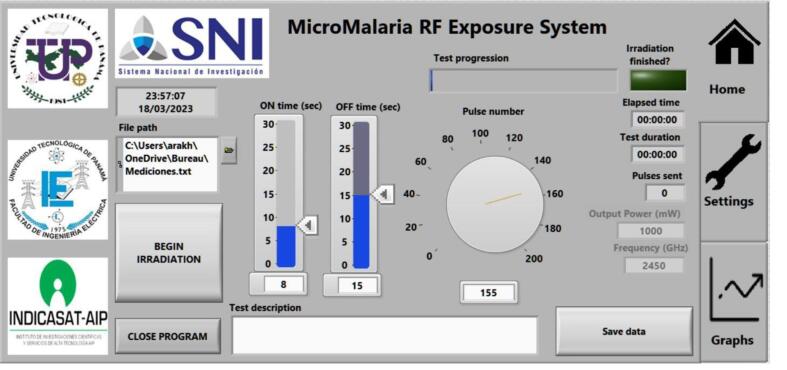
Irradiation system final graphical user interface homepage.

**Fig. 15:**
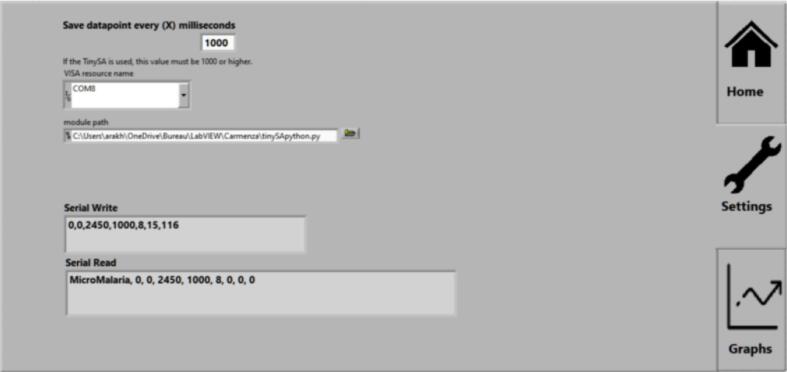
GUI settings tab.

The following descriptions refer to Fig. 14 and Fig. 15.

• ***Begin irradiation:*** *Starts the irradiation test.*
• ***Close program:*** *Closes the GUI.*
• ***ON TIME (*sec*):*** *Time in which the RF generator remains ON.*
• ***OFF TIME (*sec*):*** *Time in which the RF generator remains off.*
• ***Pulse number:*** *Number of ON/OFF cycles.*
• ***Test duration:*** *Duration of the irradiation test. It is automatically calculated by the GUI based on the duty cycle and the number of pulses.*
• ***Elapsed time:*** *Elapsed time of the irradiation test.*
• ***Test progression:*** *Progress bar that graphically shows the duration of the test.*
• ***Pulses sent:*** *Number of pulses sent throughout the test (in real time).*
• ***Output Power (mW):*** *Output power of the amplifier.*
• ***Output frequency (MHz):*** *Output frequency of the amplifier.*
• ***Test description:*** *Textbox to place description of the irradiation test to be included as a header in the measurement file (optional).*
• ***File path:*** *Path of the file with the saved measurements.*
• ***Save data:*** *Saves the data placed in the test description at the selected address.*
• ***Settings:*** *In the Settings tab, the Arduino COM port can be modified (shouldn’t be necessary as the program autodetects the Arduino device). For debugging purposes, the serial send, and the serial receive can also be seen in this tab.*
• ***Graphs:*** *If you have the recommended spectrum analyzer (TinySA Spectrum Analyzer), it is possible to observe the output power measurement through the graphical interface, by connecting and enabling the spectrum analyzer to the mini-PC through a USB port.*
• **Save datapoint every (X) milliseconds:** delay between sample recording when the save data button is enabled.
• **VISA resource name:** COM port for the Arduino communication (for advanced use since the LabVIEW code automatically detects the correct Arduino port).
• **Module path:** path containing the python script for communication with the TinySA spectrum analyzer.
• **Serial Write:** data sent through the serial port to the Arduino (debugging purposes).
• **Serial Read:** data read through the serial port from the Arduino (debugging purposes).

**Notes:**

• *After starting the irradiation test, the configuration buttons will be locked. This is intended to avoid unintentional parameter changes during the test.*
• *To close the GUI, do it through the close program button. Otherwise, communication errors might occur between the PC and the Arduino.*
• *When an irradiation test ends, the Irradiation finished? LED will turn on.*
• *In case of emergency or unexpected behavior, hit the emergency stop button located at the left of the device. Do not unplug the device directly, as it would damage the miniPC.*

To obtain the best results in irradiation tests, place the following parameters:

1. *ON TIME (*sec*): 5 s*
2. *OFF TIME (*sec*): 18 s*
3. *Pulse number: 116 pulses*

With this configuration, the highest percentages of inhibition of parasite growth are obtained (∼90 %).

## 7 Validation and characterization

The validation and characterization are presented in five different sections. Firstly, the physical and electrical specifications of the proposed device are shown. Secondly, the characterization of the electrical properties of the redesigned RF applicator, thirdly, the power and frequency validation of the RF signal output of the complete irradiation system, fourthly, a validation of the heat dissipation capacity during continuous operation and, fifthly, the validation regarding the parasite’s growth inhibition post- exposure with the device.

### 7.1 Physical and electrical specifications

The physical dimensions and the electrical specifications of the proposed device can be seen in Table 7 and Table 8.

**Table 7:** Physical dimensions and weight for the original and the proposed device.

**ORIGINAL SYSTEM**				**PROPOSED DEVICE**			
**Component**	**Weight (kg)**	**Cost (USD)**	**Volume (cm3)**	**Component**	**Weight (kg)**	**Cost (USD)**	**Volume (cm^3^)**
Keysight E4433A RF Generator	12.70	27,505.00	187,205.82	MAX2870 RF Generator	0.10	44.46	6,573.42
OPHIR 5191 RF Amplifier	16.78	12,000.00		SKY66292-11 RF Amplifier	0.12	105.19	
Narda 3003–30 Uni-Directional Coupler	0.45	263.00		ZABDC20-252H + Bi-Directional Coupler	0.24	151.73	
HP Desktop Computer	7.23	499.99		Mini PC + Touchscreen	1.65	265.98	
Stainless steel laboratory cart	15.40	560.48		Acrylic casing/ implemented electronic components	1.58	100.00	
TOTAL:	52.56	40,828.47		TOTAL:	3.69	667.36*	

*: Proposed device cost. Does not include the RF applicator.

**Table 8:** Electrical specifications of the proposed device.

**Electrical specifications**			**Unit**
Voltage	nominal	120	V
Current	standby	0.24	A
	irradiating	0.33	
Power	standby	29	W
	irradiating	40	
RF power	maximum	30.80	dBm

### 7.2 Electrical performance of the RF applicator

**Fig. 16:**
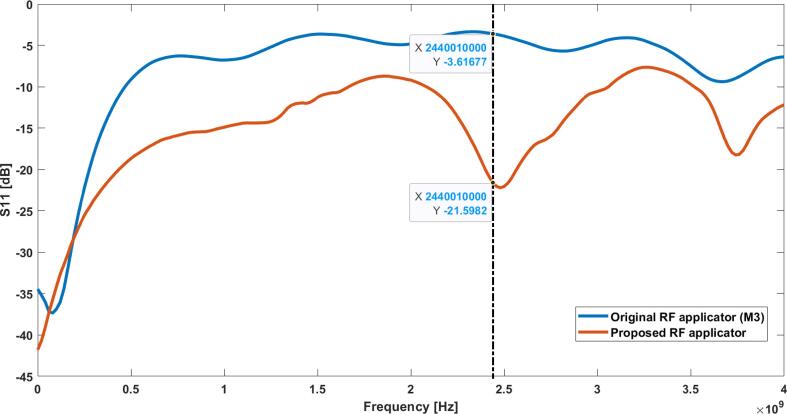
S11 comparison between the original and the new RF applicator when loaded with a blood sample. Dashed line shows values at 2.45 GHz. Measurements were taken with a FieldFox RF and Microwave Analyzer N9913A.

To validate the electrical performance of the RF applicator, the S11 coefficient was compared between the original RF applicator (M3) and the redesigned RF applicator. Measurements were taken with a FieldFox RF and Microwave Analyzer N9913A (see Fig. 16).

These results show a significant improvement in the S11 coefficient of the RF applicator compared to the original M3 applicator, when loaded with a blood sample, going from −5.21 dB to −21.6 dB.

### 7.3 Power and frequency output

**Fig. 17:**
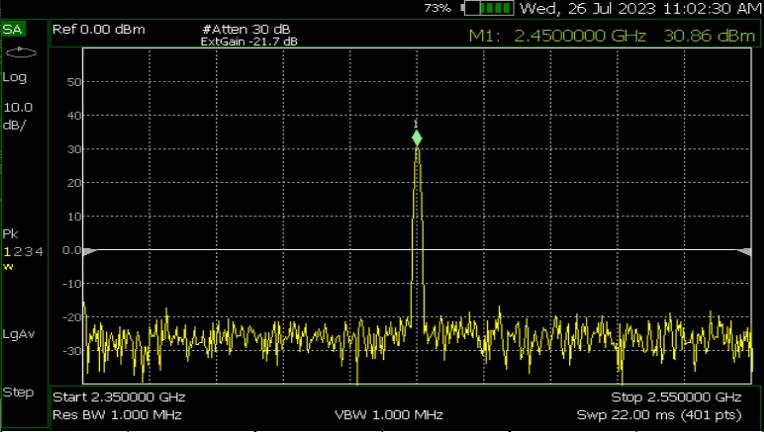
Irradiation system’s output power. Measurements were made with a FieldFox RF and Microwave Analyzer N9913A.

Fig. 17 shows the maximum output power of the proposed irradiation system. At 2.45 GHz, the output power reaches 30.86 dBm, marginally exceeding the biological protocol's stipulated power of 30 dBm. The output power can be modified using external attenuators.

### 7.4 Heat dissipation tests in continuous operation

**Fig. 18:**
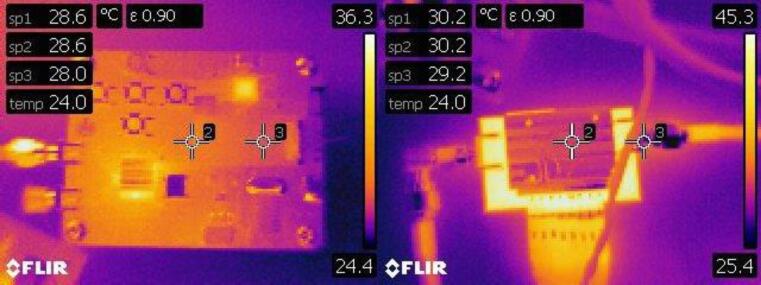
RF generator and amplifier temperature profiles after 24 h of continuous operation.

**Fig. 19:**
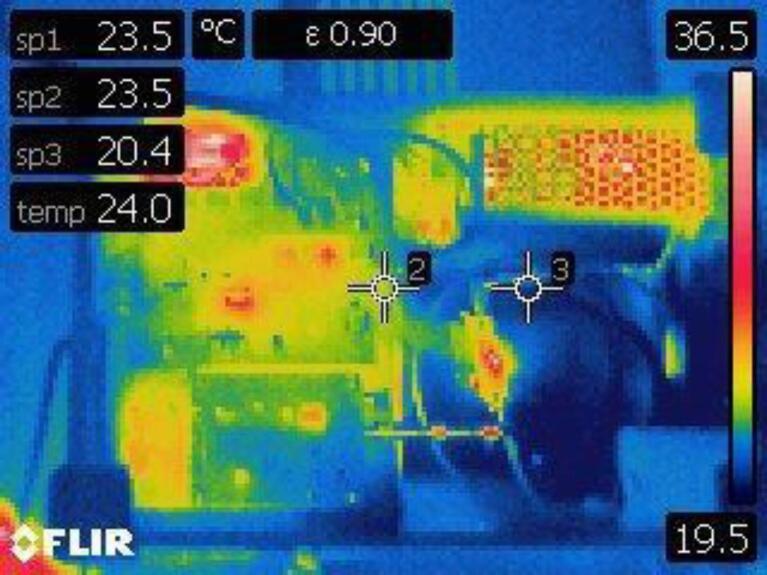
Irradiation system temperature measurement.

It is necessary for the system to work continuously for prolonged periods without overheating. For this reason, peripheral temperature measurements were made with a FLIR E4 Thermal Camera (Teledyne FLIR, OR United States). Fig. 18 and Fig. 19 show the temperature profiles of the RF generator, RF amplifier and miniaturized irradiation system respectively, after 24 h continuous of operation (at the recommended settings of 5 s ON time and 16 s OFF time) in a room at 24 °C and 60 % relative humidity.

As for the RF generator, the maximum temperature measured was 36.3° C and this corresponds to the temperature of the integrated circuit MAX2870, which can work at temperatures of up to 85 °C. The RF amplifier SKY66292-11 presented a temperature of 30.2 °C in its heatsink and 45.3° in its circuitry. These values are considered stable as the maximum operating temperature of the amplifier is 100 °C. Finally, a capture of the internal circuitry of the irradiation system was taken. The hottest points correspond to the filtering capacitors of the power supply, the step-down converter to 5 V DC and the integrated circuit of the amplifier. Despite this, the maximum temperature in this capture was 36.5 °C.

### 7.5 Biological validation: Parasite growth inhibition

**Fig. 20:**
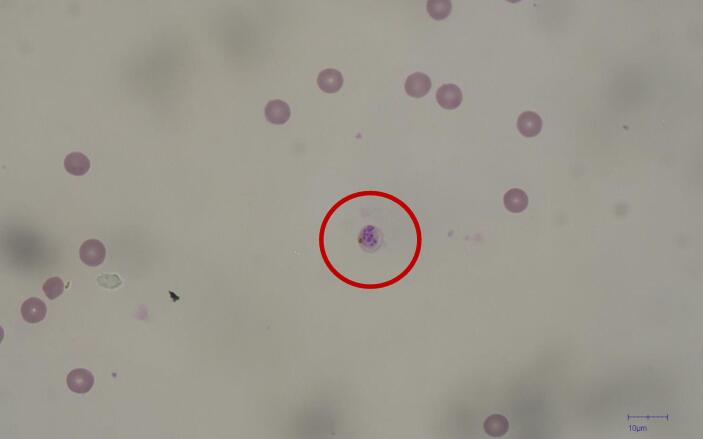
Microscope imaging depicting a schizont before microwave exposure. Images were taken with an Olympus IX83 (Olympus Corporation, Tokyo, Japan).

The biological validation was performed using the same protocol described in the original study [5]. The process regarding parasite culture preparation is beyond the scope of this work. Briefly, the protocol requires blood samples to be at 2 % parasitemia (i.e., 2 % of blood cells infected with Plasmodium parasites) and parasites to be in the early-schizont stage, as seen in Fig. 20.

**Fig. 21:**
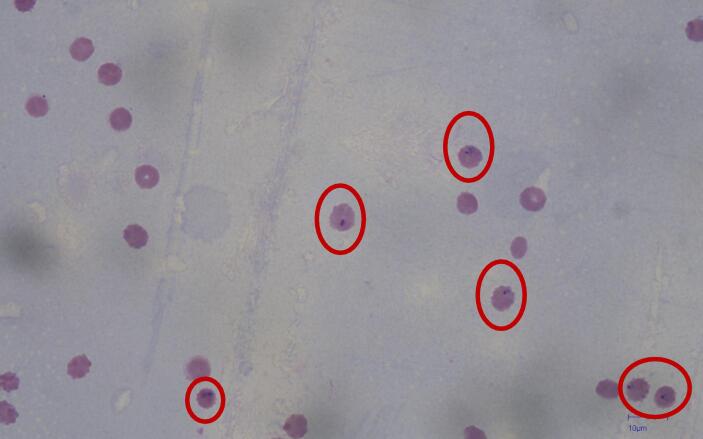
Untreated sample: merozoites infecting new erythrocytes. Images were taken with an Olympus IX83 (Olympus Corporation, Tokyo, Japan).

In the schizont stage of Plasmodium, the parasite undergoes a process involving multiple rounds of nuclear division without cell division. Eventually, this leads to the formation of numerous merozoites within the schizont. When the schizont fully matures, it causes the host erythrocyte to burst, releasing these merozoites into the bloodstream. These merozoites can then infect other erythrocytes, perpetuating the cycle of infection. Considering that the protocol states that the parasites need to be in the early-schizont stage, to assess the effectiveness of the treatment, the microscopist evaluated if the parasites continued their infection cycle (merozoites infecting other erythrocytes, as shown in Fig. 21) or if the infection cycle stopped (with infected erythrocytes still in their schizont stage, as shown in Fig. 20).

To obtain statistically significant results, the protocol required counting at least 1,000 erythrocytes, quantifying the infected and uninfected cells. Since each image captured by the microscope is not representative of the whole batch, the microscopist manually displaces the microscope slide and manually counts the infected and uninfected erythrocytes. If all infected erythrocytes were still schizonts (as at the beginning of the test), that would mean a 100 % growth inhibition effectiveness, whereas if all infected erythrocytes were infected with merozoites (as in Fig. 21), it would mean 0 % inhibition effectiveness. To ensure objectivity, the microscopist was unaware of the sample's experimental treatment.

To validate the equivalence between both systems, four irradiation tests were made. Two tests were performed to compare the M3 (the original RF applicator) and the RF applicator proposed in this work. Two other experiments were conducted to establish the equivalence between the original and the proposed system. The results for the biological validation tests are resumed in Table 9 and Table 10.

**Table 9:** Biological validation: RF applicator comparison.

**RF applicator validation**			
**Test conditions: 30 dBm, 35 % duty cycle, 2 % parasitemia**			
**System**	**Substrate cutout height (mm)**	**Microstrip width (mm)**	**Relative growth**
Control	NA	NA	100 %
M3 applicator	1.5	3	11.93 %
Proposed applicator	3	6	9.54 %

**Table 10:** Biological validation: Irradiation system comparison.

**Irradiation system validation**			
**Test conditions: 30 dBm, 35 % duty cycle, 2 % parasitemia**			
**System**	**Device Type**	**Model**	**Growth inhibition**

Control	NA	NA	17 %
Original	RF Generator	HP E4433A	91.37 %
	RF Amplifier	OPHIR 5171FE	
Proposed	RF Generator	MAX2870	89.45 %
	RF Amplifier	SKY66292-11	

These results show that the proposed irradiation device and the RF applicator are equivalent to their counterparts in terms of parasite growth inhibition.

## 8 Ethics statements

All blood donors to keep P. falciparum culture in vitro, gave their informed consent for inclusion before their blood was drawn. The study was conducted in accordance with the Declaration of Helsinki, and the protocol was approved by the Ethics Committee of Universidad Santa María La Antigua (Project identification code CBI-USMA 2023-P060).

Funding: The research was self-funded by the participating institutions, and no external agencies or grant numbers are associated with the support of this study.

## 9 Declaration of Generative AI and AI-assisted technologies in the writing process

During the preparation of this work the authors used ChatGPT to improve readability. After using this tool/service, the authors reviewed and edited the content as needed and take full responsibility for the content of the publication.

## CRediT authorship contribution statement

**Esteban Rua:** Data curation, Investigation, Methodology, Software, Validation, Writing – original draft. **Lorena Coronado:** Investigation, Validation, Visualization. **Carlos A. Donado Morcillo:** Visualization, Investigation. **Ricardo Correa:** Supervision, Validation. **Lina Solís:** Software, Validation. **Carmenza Spadafora:** Data curation, Funding acquisition, Resources. **Alejandro Von Chong:** Conceptualization, Funding acquisition, Investigation, Methodology, Project administration, Resources, Software, Supervision, Visualization, Writing – review & editing.

## Declaration of competing interest

The authors declare that they have no known competing financial interests or personal relationships that could have appeared to influence the work reported in this paper.
